# Establishment of a new initial dose plan for vancomycin using the generalized linear mixed model

**DOI:** 10.1186/s12976-017-0054-9

**Published:** 2017-04-08

**Authors:** Yasuyuki Kourogi, Kenji Ogata, Norito Takamura, Jin Tokunaga, Nao Setoguchi, Mitsuhiro Kai, Emi Tanaka, Susumu Chiyotanda

**Affiliations:** 1 Chiyoda Hospital, Social Medial Corporation Senwakai, Hyuga, Japan; 2 School of Pharmaceutical Sciences, Kyushu University of Health and Welfare, Nobeoka, Japan; 3 Second Department of Clinical Pharmacy, Graduate School of Clinical Pharmacy, Kyushu University of Health and Welfare, 1714-1 Yoshino, Nobeoka, Miyazaki 882-8508 Japan

**Keywords:** Vancomycin, Therapeutic drug monitoring, Initial dose planning, Generalized linear mixed model

## Abstract

**Background:**

When administering vancomycin hydrochloride (VCM), the initial dose is adjusted to ensure that the steady-state trough value (Css-trough) remains within the effective concentration range. However, the Css-trough (population mean method predicted value [PMMPV]) calculated using the population mean method (PMM) often deviate from the effective concentration range. In this study, we used the generalized linear mixed model (GLMM) for initial dose planning to create a model that accurately predicts Css-trough, and subsequently assessed its prediction accuracy.

**Methods:**

The study included 46 subjects whose trough values were measured after receiving VCM. We calculated the Css-trough (Bayesian estimate predicted value [BEPV]) from the Bayesian estimates of trough values. Using the patients’ medical data, we created models that predict the BEPV and selected the model with minimum information criterion (GLMM best model). We then calculated the Css-trough (GLMMPV) from the GLMM best model and compared the BEPV correlation with GLMMPV and with PMMPV.

**Results:**

The GLMM best model was {[0.977 + (males: 0.029 or females: -0.081)] × PMMPV + 0.101 × BUN/adjusted SCr – 12.899 × SCr adjusted amount}. The coefficients of determination for BEPV/GLMMPV and BEPV/PMMPV were 0.623 and 0.513, respectively.

**Conclusion:**

We demonstrated that the GLMM best model was more accurate in predicting the Css-trough than the PMM.

## Background

**Fig. 1:**
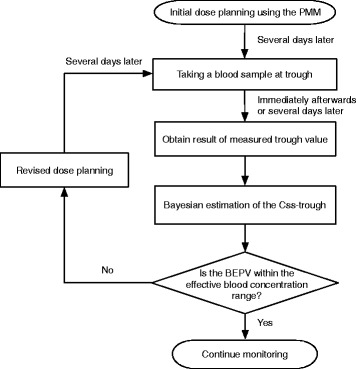
TDM protocol for VCM

Vancomycin hydrochloride (VCM) is commonly used to treat methicillin-resistant *Staphylococcus aureus* (MRSA) infections but is known to have a narrow safe blood concentration range. To ensure safe and effective pharmacotherapy, the steady-state trough value (Css-trough) must be maintained at the effective blood concentration range of 10–20 μg/mL [1, 2]. The incidence of renal toxicity is known to increase when the Css-trough exceed 20 μg/mL [3, 4]. Therefore, the VCM dose must be adjusted using therapeutic drug monitoring (TDM) to keep the Css-trough within the effective blood concentration range (Fig. 1). This improves the cure rate of infections and the incidence of renal toxicity [5]. Because VCM has a high rate of renal excretion, the dose setting must be determined by the renal function of the patient. Therefore, the initial dose plan for VCM is set using the population mean method (PMM), which uses mean values for population pharmacokinetics parameters, creatinine clearance (CLcr) and weight to estimate the Css-trough (population mean method predicted value, PMMPV). Then, the estimated Css-trough is used to determine the VCM dose and infusion time and interval that would maintain an effective blood concentration range. VCM administration is commenced based on this initial dose plan and after several days the trough value is measured. We calculate the Css-trough (Bayesian estimate predicted value, BEPV) from the Bayesian estimate using the measured value and the population pharmacokinetics parameters. There is a large discrepancy between the PMMPV and BEPV in cases where the accuracy of the Css-trough determined from the initial dose plan as predicted by PMM is low. In that case, BEPV often deviates from the effective blood concentration range. Because the dose plan has to be changed in such cases, it requires sufficient time that Css-trough achieves the effective blood concentration range. As a result, the duration of infection is prolonged, the risk of adverse effects is higher. Therefore, in initial dose plan, it is necessary to devise to predict highly accurate Css-trough. However, because the predictive accuracy by the PMM is insufficient, a large discrepancy often exists between the PMMPV and BEPV [6].

**Fig. 2:**
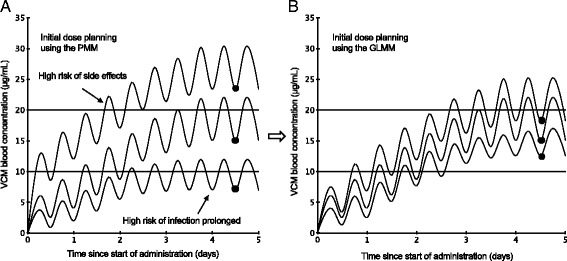
Changes in blood concentration after start of VCM administration. Changes in VCM blood concentration when initial dose planning is performed using (**a**) PMM and (**b**) GLMM. ●, Css-trough. The purpose of this study was to create the model that accurately predicts Css-trough at the initial VCM dose plan

PMM is a method of predicting the unknown Css-trough before commencing drug administration. Statistical modeling has attracted attention as a method of predicting unknown results using a formula (model) created by extracting only the necessary information from enormous amounts of data and is used in a variety of fields [7, 8]. One statistical modeling method is the generalized linear mixed model (GLMM), which is characterized by its ability to use multiple data (explanatory variables) to predict unknown outcomes (response variables). Medical facilities accumulate a variety of medical data, but when PMM is used to determine the initial VCM dose, only medical data such as the CLcr can be used. Therefore, we extracted that type of information that had a major impact on changes of Css-trough of VCM from patient’s medical data and created a model that predicts highly accurate Css-trough by applying that information to GLMM explanatory variable (Fig. 2). In this study, we created this model and assessed whether it could predict VCM Css-trough values that were closer to the BEPV than PMMPV, calculated using the model.

## Methods

### Subject extraction

This study included 46 patients whose trough values were measured in 3–5 days from the start of drug administration and were selected from patients who received VCM (VANCOMYCIN HYDROCHLORIDE for I.V. Infusion “MEEK”, Meiji Seika Pharma, Tokyo, Japan) drip infusions between August 2008 and March 2015 at Chiyoda Hospital (Table 1). Exclusion criteria were receiving hemodialysis, outpatients, and under the age of 18 years.

**Table 1:** Summary of patient characteristics

Characteristic	
No. of patients (female/male)	46 (14/32)
Age (years)	77.37 ± 8.79
Height (cm)	157.66 ± 8.59
Weight (kg)	46.66 ± 9.91
BMI (kg/m^2^)	18.70 ± 3.34
SCr (mg/dL)	0.82 ± 0.35
CLcr (mL/min)	45.37 ± 18.31
BUN (mg/dL)	19.15 ± 11.76
AST (IU/L)	34.70 ± 24.64
ALT (IU/L)	30.46 ± 36.94
CRP (mg/dL)	8.98 ± 7.32

The values are shown as the mean ± standard deviation

### Calculation of CLcr

The PMM requires the CLcr for calculating the VCM Css-trough values and, therefore, we first calculated the CLcr for each patient from their sex, age, weight, and serum creatinine (SCr) at initial dose planning using the Cockcroft-Gault formula (CG formula, Eq. 1) [9]. SCr was affected by the patient’s muscle mass. Therefore, because patients with low muscle mass have low SCr levels, we estimate that the CLcr calculated using the CG formula would high, which overestimates the renal function. In Japan, to estimate CLcr calculated using the CG formula accurately, if the patient’s SCr is < 0.6 mg/dL, it is commonly adjusted to 0.6 mg/dL (adjusted SCr) [10]. Therefore, we used the same method here.

*Women*

1A $$ \mathrm{CLcr}\ \left(\mathrm{mL}/ \min \right) = \frac{\left[140-\mathrm{Age}\ \left(\mathrm{years}\right)\right] \times \mathrm{Weight}\ \left(\mathrm{kg}\right)}{72 \times \mathrm{SCr}\ \left(\mathrm{mg}/\mathrm{dL}\right)} \times 0.85 $$

*Men*

1B $$ \mathrm{CLcr}\ \left(\mathrm{mL}/ \min \right) = \frac{\left[140-\mathrm{Age}\ \left(\mathrm{years}\right)\right] \times \mathrm{Weight}\ \left(\mathrm{kg}\right)}{72 \times \mathrm{SCr}\ \left(\mathrm{mg}/\mathrm{dL}\right)} $$

To calculate BEPV, the CLcr is calculated using Eq. 1 even when trough values are measured. If the SCr level has not reached 0.6 mg/dL then, it is adjusted accordingly.

### Calculation of PMMPV and BEPV

The PMMPV was calculated using CLcr at initial dose planning, weight, VCM dose conditions (dose, infusion time, and administration interval), and mean values for population pharmacokinetic parameters [11]. Furthermore, the calculations were based on the two-compartment model because the distribution of VCM is divided into the central compartment (blood and tissues which equilibrate rapidly with blood) and the peripheral compartment (tissues which equilibrate slowly with blood) [11].

The BEPV was calculated by CLcr at measuring trough value, weight, VCM administration conditions (dose, infusion time, and administration interval), and the estimating the patients’ pharmacokinetic parameters based on the two-compartment model using the Bayesian estimate.

The PMMPV and BEPV were calculated using the TDM analytical software, Vancomycin MEEK Ver. 3.0 (Meiji Seika Pharma).

### Definition of difference (PMM prediction deviation quantity, PMMPDQ) between BEPV and PMMPV

This study aimed to create a model that very accurately predicts the VCM Css-trough using patient medical data. We focused on the medical data having high correlation with the difference between BEPV and PMMPV. We could reduce the difference between BEPV and PMMPV by applying the medical data to the GLMM model as explanation variables. Thus, the difference between BEPV and PMMPV is defined as the PMM prediction deviation quantity (PMMPDQ, Eq. 2).

2 $$ \mathrm{PMMPDQ} = \mathrm{BEPV} - \mathrm{PMMPV} $$

### Establishing the basic model that the aimed model is based on

Before creating the model, we first established the minimum configuration model (basic model) that formed its basis. Here, we attempted to use a model to very accurately predict the BEPV. Therefore, the response variable used in the model was the BEPV. Our investigation of the correlation between PMMPV and BEPV indicated that it was 0.702 (Spearman’s rank correlation coefficient). Guilford’s rule of thumb, which is commonly used as a standard for correlation coefficients, stipulates that correlation coefficients of 0–0.2, 0.2–0.4, 0.4–0.7, 0.7–0.9, and 0.9–1.0 are “almost none,” “weak,” “moderate,” “high,” and “extremely high” correlations, respectively [12]. Therefore, since PMMPV and BEPV are highly correlated, we believe that PMMPV is an appropriate explanatory variable for the model. Based on this, the basic model is expressed as Eq. 3 and is equivalent to the formula that predicts the Css-trough based on PMM.

3 $$ \mathrm{BEPV}={\upbeta}_1 \times \mathrm{PMMPV} $$

β_1_: PMMPV coefficient

### Creating the predictor model (GLMM best model) for VCM Css-trough based on GLMM

**Fig. 3:**
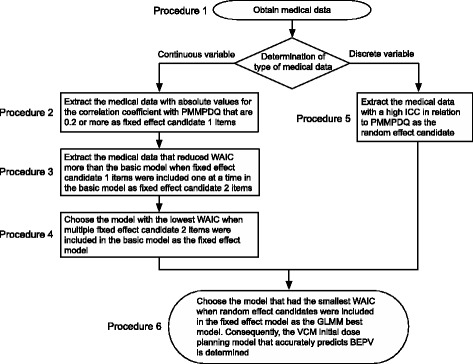
Procedure for creating GLMM best model for estimating Css-trough of VCM based on WAIC. Models with small WAIC have small predictive errors. We used WAIC to extract effective medical data (fixed and random effects) and determined the model that accurately predicts BEPV (GLMM best model)

Figure 3 shows the procedure we used to create the model (GLMM best model). To create a model with a high predictive accuracy, it is necessary to add effective explanatory variables to the basic model. The use of medical data that is highly correlated to PMMPDQ in the model improves the predictive accuracy. Therefore, the explanatory variables added to the basic model must be medical data that is highly correlated to PMMPDQ. Thus, to identify medical data as potential explanatory variables that can be added to the basic model, we first obtained the subjects’ medical data.

### Collection of subject medical data (Fig. 3, procedure 1)

To obtain medical data that can potentially be added to the basic model as explanatory variables, we collected the following subject data: Clinical findings (age, age range [10-year intervals], aged ≥ 75 or not, sex, height, weight, hospital days since drug administration commenced), blood test findings (total protein, serum albumin [Alb], aspartate transaminase [AST], alanine transaminase [ALT], lactate dehydrogenase [LDH], total bilirubin, blood urea nitrogen [BUN], SCr, adjusted SCr, BUN/SCr, BUN/adjusted SCr, SCr adjustment amount, SCr adjusted or not, serum Na, serum K, serum Cl, blood glucose level, c-reactive protein [CRP], white blood cell [WBC], red blood cell [RBC], hemoglobin [Hb], hematocrit [Ht], platelet [PLT], mean corpuscular volume [MCV], mean corpuscular hemoglobin [MCH], mean corpuscular hemoglobin concentration [MCHC]), and VCM administration schedule (initial dose, initial daily dose, single dose, daily dose, infusion time, and number of doses; whether doses were irregularly spaced; and number of days until blood concentration trough values were measured since drug administration commenced). Then, to extract effective explanatory variables from the medical data, we conducted the following investigation.

### Extraction of medical data (fixed effect candidate 1) correlated to the difference between BEPV and PMMPV (PMMPDQ, Fig. 3, procedure 2)

When using GLMM, multiple explanatory variables can be included in the model. However, the creation of a model including all the medical data we obtained would have produced an inordinate number of model types. Therefore, we first extracted the patient medical data (explanatory variables) that would be effective when added to the basic model. Thus, the two GLMM explanatory variables types were the fixed effect (equivalent to single and multiple regression analyses explanatory variables), which were elements that predict BEPV (response variable), and random effect, which were elements that changed the fixed effect coefficient and the intercept values of the model. In accordance with the software specifications (Stan) used for the GLMM analysis, we used continuous variables (continuous, such as height and weight) for the fixed effect and discrete variables (qualitatively non-continuous, such as sex and all conditions) for random variables.

Since the medical data that correlated highly with the PMMPDQ had an appropriate fixed effect for use in the model, we calculated the Spearman’s rank correlation coefficient for all medical data and the PMMPDQ.

Medical data (continuous variable) that correlated highly to the PMMPDQ (absolute value of the correlation coefficient of ≥ 0.2) were identified as fixed effect candidate 1.

### Extraction of appropriate medical data (fixed effect candidate 2) for use as fixed effect in model (Fig. 3, procedure 3)

We wanted to extract medical data from items identified as fixed effect candidate 1 that would be effective when added to the basic model (Eq. 3). Therefore, we created a model (Eq. 4) that included each fixed effect candidate 1 item as a fixed effect in the basic model and calculated the information criterion (Widely Applicable Information Criterion, WAIC) for each model.

4 $$ \mathrm{BEPV} = {\upbeta}_1 \times \mathrm{PMMPV} + {\upbeta}_{\mathrm{FE}1} \times \mathrm{F}\mathrm{E}1 $$

β_1_: PMMPV (fixed effect) coefficient, β_FE1_: each fixed effect candidate 1 (fixed effect) coefficients, and FE1: each fixed effect candidate 1 (fixed effect).

WAIC is used to select the model with a high degree of predictive accuracy from multiple models and is an index for generalization errors (predictive error when making predictions using the model on unknown patients other than the subjects of this study). The smaller WAIC is, the higher predictive accuracy of a model is and it is determined to apply to unknown patients [13]. Therefore, fixed candidate 1 items used in a model made smaller WAIC than the basic model were considered an appropriate fixed effect in the GLMM best model and were designated as fixed effect candidate 2.

### Determination of fixed effect model (Fig. 3, procedure 4)

To determine the model (fixed effect model) composed of multiple fixed effects with the smallest predictive error, we created a model (Eq. 5) that included multiple fixed effect candidate 2 items in the basic model. Additionally, we calculated the WAIC for all models.

5 $$ \mathrm{BEPV}={\upbeta}_1 \times \mathrm{PMMPV} + {\displaystyle \sum_{i=1}^n{\beta}_{\mathrm{FE}2 i}} \times \mathrm{F}\mathrm{E}2 i $$

β_1_: PMMPV (fixed effect) coefficient, β_FE2*i*_: *i*th fixed effect candidate 2 coefficient, and FE2*i*: *i*th fixed effect candidate 2 (fixed effect).

However, *n* is the upper limit of the number of medical data items corresponding to fixed effect candidate 2.

Of all the models created, that with the smallest WAIC was selected as the fixed effect model.

### Extracting applicable medical data (random effect candidate) as random effect in the model (Fig. 3, procedure 5)

Since medical data that is highly correlated to the PMMPDQ has a major effect on predictive accuracy, we calculated the intra-class correlation coefficient (ICC). Since medical data with a large ICC related to the PMMPDQ is a likely discrete variable that can be applied to the model [14], we identified the medical data (discrete variables) with the largest ICC as random effect candidates.

### Determination of GLMM best model (Fig. 3, procedure 6)

To determine the most appropriate predictive model (GLMM best model) with the smallest predictive error, we created multiple models including random effect candidate items in the fixed effect model (the model created using Procedure 4 in Fig. 3) and calculated WAIC for each model. Of the created models, that with the smallest WAIC was selected as the GLMM best model.

### Assessing predictive accuracy of GLMM best model

First, we substituted the subjects’ medical data for all the explanatory variables (fixed and random effects) in the GLMM best model, which we used to calculate the Css-trough (GLMMPV). Next, to assess the predictive accuracy of the PMMPV and GLMMPV for BEPV, we set BEPV as the response variable and investigated the regression equation and coefficient of determination (R^2^) when the explanatory variable was either PMMPV or GLMMPV. The GLMM prediction deviation quantity (GLMMPDQ) was defined as the difference between BEPV and GLMMPV (Eq. 6).

6 $$ \mathrm{GLMMPDQ} = \mathrm{BEPV} - \mathrm{GLMMPV} $$

### Data processing method

We used the statistical analysis software R (ver. 3.2.3) and Microsoft Excel for Mac (ver. 15.22) to statistically analyze the data. We used functions included in R for our Spearman’s rank correlation coefficient calculations and Shapiro-Wilk test and the R package ICC (ver. 2.3.0) for ICC calculations. We used Excel for Mac for simple linear regression and R^2^ calculations. A P <0.05 was considered significant for all tests.

We used R, Stan, the R packages rstan, and brms (ver. 2.9, 2.9.0-3, and 0.8.0, respectively) for GLMM analysis and WAIC calculations. We used the Bayesian estimation with Hamilton Monte Carlo to estimate the model coefficient. We used Rhat for the convergence test of the Bayesian estimation and determined that its convergence with Rhat was ≤ 1.1 [15]. The settings of brm function in brms package were as follows: Chains = 3, Iter = 30000 (100000 when random variables were included in the model), Warmup = 15000 (50000 when random variables were included in the model), Thin = 2, and Family = “normal.” When using the Shapiro-Wilk test on the BEPV, the null hypothesis that followed the normal distribution was not rejected (*P* = 0.19). Thus, the probability distribution for the response variable was a normal distribution.

## Results

This study aimed to create a model (GLMM best model) that highly accurately predicts the Css-trough of the initial dose plan for VCM using patient medical data in the GLMM. Additionally, we assessed whether the VCM Css-trough values (GLMMPV) calculated using the GLMM best model were closer to the BEPV than the PMMPV. First, because we thought the medical data correlating to the difference (PMMPDQ) between BEPV and PMMPV would decrease the predictive error, we extracted the medical data (fixed effect candidate 1) that was highly correlated with the PMMPDQ. Next, to extract the medical data that could be applied to the GLMM best model, we created a model (Eq. 4) including each the fixed effect candidate 1 item in the basic model (Eq. 3). Then, we selected the medical data (fixed effect candidate 2) that made the WAIC of the model smaller (the smaller the WAIC, the higher the predictive accuracy of the model and the smaller the generalization error). Then, we created a model (Eq. 5) that included multiple fixed effect candidate 2 items in the basic model and selected the model with the smallest WAIC as the fixed effect model. We designated the medical data with the largest PMMPDQ-related ICC as the random effect candidate items. We created multiple models that included the random effect candidate items in the fixed effect model and selected the model with the smallest WAIC as the GLMM best model. Finally, in to assess the GLMMPV accuracy, we investigated the simple linear regression and R^2^ when the response variable was BEPV and the explanatory variable was either the PMMPV or the GLMMPV. Details of the results are below.

### GLMM best model construction

#### Extraction of medical data (fixed effect candidate 1) that correlated with the difference (PMMPDQ) between BEPV and PMMPV (Fig. 3, procedure 2)

To increase the predictive accuracy of Css-trough when setting the VCM initial dose plan, the difference between BEPV and PMMPV, which is the absolute value of the PMMPDQ (Eq. 2), had to be reduced. Because the medical data items that correlated highly with the PMMPDQ had a large effect on predictive accuracy, their inclusion in the model would allow the predictive deviation to be reduced (that is, increase predictive accuracy). First, we investigated the correlation between the PMMPDQ and all the medical data items (continuous variables). The results indicated that 10 types of medical data with absolute correlation coefficient values with the PMMPDQ of ≥ 0.2 (BUN/adjusted SCr, BUN, BUN/SCr, AST, Age, SCr, CLcr, SCr amount adjusted, single dose, and daily dose, Table 2) were factors with a major effect on predictive accuracy. To determine whether they could be used as fixed effect items in the model we created, we conducted the following investigations on the 10 types of medical data as fixed effect candidate 1 items.

**Table 2:** Correlation coefficient for fixed effect candidate 1 and PMMPDQ

Fixed effect candidate 1 (medical data)	Correlation coefficient	*p*-value
BUN/adjusted SCr	0.398	0.006^*^
BUN	0.372	0.011^*^
BUN/SCr	0.332	0.024^*^
AST	0.253	0.090
Age	0.248	0.096
SCr	0.215	0.152
CLcr	-0.233	0.119
SCr adjusted amount	-0.239	0.110
Single dose	-0.263	0.078
Daily dose	-0.279	0.060

Asterisks indicate *p* < 0.05

#### Extracting medical data (fixed effect candidate 2) that was applicable as fixed effect (Fig. 3, procedure 3)

To further extract the medical data that was applicable to the model from the fixed effect candidate 1 items, we created a model (Eq. 4) that included each of the fixed effect candidate 1 items in the basic model (Eq. 3) and calculated WAIC. Declines in WAIC indicate a reduced prediction error. Of the models with the fixed effect candidate 1, the one with a smaller WAIC than the basic model used the following medical data: BUN/adjusted SCr, BUN, BUN/SCr, age, and SCr adjusted amount (Table 3). There is a high probability that these medical data items can be used as an applicable fixed effect for the GLMM best model. Because the BUN/adjusted SCr and BUN/SCr are similar parameters, we used only the BUN/adjusted SCr with a low WAIC value. Therefore, we used the BUN/adjusted SCr, BUN, age, and SCr adjusted amount as the fixed effect candidate 2 items in the following investigation.

**Table 3:** WAIC and the Coefficients of the variables when all fixed effect candidate 1 items are included in basic model

Fixed effect candidate 1 (medical data)	Coefficient (l-95% CI, u-95% CI)	WAIC
None (Basic model)	-	258.42
BUN/adjusted SCr	0.1 (0.02, 0.17)	254.52^a^
BUN	0.09 (0.01, 0.17)	256.12^a^
BUN/SCr	0.08 (0.00, 0.16)	256.15^a^
AST	0.01 (-0.03, 0.05)	260.46
Age	0.04 (-0.01, 0.10)	257.51^a^
SCr	1.13 (-1.48, 3.78)	259.73
CLcr	-0.01 (-0.07, 0.04)	260.6
SCr adjusted amount	-16.09 (-32.54, 0.26)	256.18^a^
Single dose	0.00 (-0.01, 0.00)	260.6
Daily dose	0.00 (0.00, 0.00)	259.63

^a^WAIC of the model (Ep. 4) that included fixed effect candidate 1 item was smaller than the WAIC of the basic model (Ep. 3). Smaller WAIC indicates decreased predictive error in the model

#### Fixed effect model determination (Fig. 3, procedure 4)

The GLMM model can use multiple fixed effect items. Therefore, we created a model (Eq. 5) that included multiple fixed effect candidate 2 items in the basic model (Eq. 3) and calculated the WAIC. The results indicate that the WAIC of the model that simultaneously included BUN/adjusted SCr and SCr adjusted amount was the lowest (253.45, Table 4). Based on this, we designated this model as the fixed effect model.

**Table 4:** WAIC when multiple fixed effect candidate 2 are included in the basic model

Fixed effect candidate 2 (medical data)	WAIC
None (Basic model)	258.42
BUN/adjusted SCr and BUN	254.94
BUN/adjusted SCr and SCr adjusted amount	253.45^a^
BUN/adjusted SCr and Age	256.26
BUN and SCr adjusted amount	254.33
BUN and Age	256.05
SCr adjusted amount and Age	256.03
BUN/adjusted SCr and BUN and SCr adjusted amount	254.58
BUN/adjusted SCr and BUN and Age	256.83
BUN/adjusted SCr and SCr adjusted amount and Age	255.25
BUN and SCr adjusted amount and Age	256.14
BUN/adjusted SCr and BUN and SCr adjusted amount and Age	256.56

^a^Lowest WAIC above. Lower WAIC indicates decreased predictive error in the model

#### Extracting medical data (random effect) that was applicable as random effect (Fig. 3, procedure 5)

Because medical data items (discrete variables) with a large PMMPDQ-related ICC affect the predictive accuracy considerably, it is highly likely that they can be used as applicable random effect items in the GLMM best model. Thus, we calculated the ICC for all PMMPDQ-related medical data items (Table 5). The item with the largest PMMPDQ-related ICC was sex (0.057) and, therefore, it was used as the random effect candidate item in the following investigation.

**Table 5:** ICC for medical data (discrete variables) related to PMMPDQ

Medical data (discrete variables)	ICC	l-95% CI	u-95% CI
Sex	0.057	-0.032	0.991
Adjusted SCr	0.036	-0.041	0.989
Aged 75 or above	0.023	-0.039	0.987
No. of days from start of administration to blood test for blood concentration trough	-0.043	-0.065	0.484
Age group (10-year intervals)	-0.044	-0.117	0.392
Irregular interval administration	-0.047	-0.047	-0.037
No. of doses	-0.071	-0.140	0.358

Medical data (discrete variables) with a large ICC in relation to PMMPDQ have a high likelihood of being random effect items suitable for use in the GLMM best model

#### GLMM best model determination (Fig. 3, procedure 6)

To determine the optimum predictive model (GLMM best model) that reduces prediction error the most and includes fixed and random effects, we created multiple models that included sex (random effect) in the fixed effect model (the model determined using Procedure 4 in Fig. 3) and calculated WAIC (Table 6). The results indicated that the model including sex (random effect) in the PMMPV (fixed effect) coefficient had the smallest WAIC (252.01). Therefore, we designated this model as the GLMM best model.

**Table 6:** WAIC when random effects are included in the fixed effect model

Fixed effect including random effect (Sex)	WAIC
None (fixed effect model)	253.45
PMMPV	252.01^a^
BUN/adjusted SCr	252.29
SCr adjusted amount	252.34
PMMPV and BUN/adjusted SCr	253.65
PMMPV and SCr adjusted amount	253.39
BUN/adjusted SCr and SCr adjusted amount	252.69
PMMPV and BUN/adjusted SCr and SCr adjusted amount	253.11

^a^Lowest WAIC above. Lower WAIC indicates decreased predictive error in the model

Based on the above results, we determined the GLMM best model (Eq. 7) would predict the Css-trough with high accuracy when establishing the VCM initial dose plan.

*Women*

7A $$ \begin{array}{l}\mathrm{GLMMPV} = \left(0.977-0.081\right) \times \mathrm{PMMPV} + 0.101 \times \mathrm{BUN}/\mathrm{adjusted}\ \mathrm{SCr} - 12.899\ \\ {} \times \mathrm{SCr}\ \mathrm{adjusted}\ \mathrm{amount}\ \end{array} $$

*Men*

7B $$ \begin{array}{l}\mathrm{GLMMPV} = \left(0.977+0.029\right) \times \mathrm{PMMPV} + 0.101 \times \mathrm{BUN}/\mathrm{adjusted}\ \mathrm{SCr} - 12.899\ \\ {} \times \mathrm{SCr}\ \mathrm{adjusted}\ \mathrm{amount}\end{array} $$

The coefficients and their credible intervals (CIs) for all explanatory variables (fixed and random effects) in the GLMM best model are shown in Table 7.

**Table 7:** All explanatory variables for the GLMM best model and their coefficient

Explanatory variables	Coefficient	l-95% CI	u-95% CI
PMMPV (fixed effect)	0.977	0.314	1.960
BUN/adjusted SCr (fixed effect)	0.101	0.020	0.180
SCr adjusted amount (fixed effect)	-12.899	-28.700	2.652
Sex: Female (random effect)	-0.081	-1.201	0.592
Sex: Male (random effect)	0.029	-1.123	0.711

### Assessing predictive accuracy of GLMM best model

**Fig. 4:**
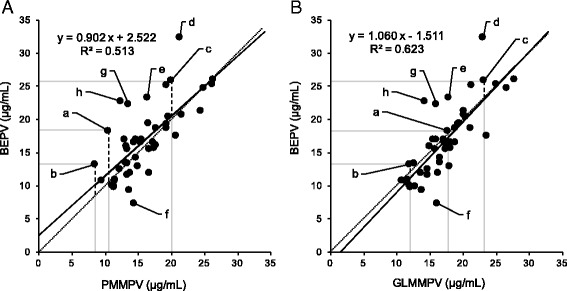
Comparison of correlation of PMMPV with BEPV and GLMMPV with BEPV. Fig. 4a indicates correlation of PMMPV and BEPV. Fig. 4b indicates correlation of GLMMPV and BEPV. Solid line is regression line with response variable as BEPV and explanatory variable as either PMMPV or GLMMPV. If Css-trough can be accurately predicted when establishing the initial dose plan, then all data plots will be located on the dotted line, and the solid and dotted lines will be identical. The letters a, b, and c indicate patients who showed improved accuracy in their VCM Css-trough predictions with the GLMM best model. The length of the dashed lines drawn vertically from a, b, and c indicates (**a**) PMMPDQ (the difference between PMMPV and BEPV) and (**b**) GLMMPDQ (the difference between GLMMPV and BEPV). The letters d, e, f, g and h indicate patients who showed the largest positive or negative deviation in their predictions

First, we investigated the simple linear regression and R^2^ when the response variable was BEPV, and the explanatory variable was either PMMPV or GLMMPV to assess the BEPV-related accuracy of PMMPV and GLMMPV. The single linear regression slope for PMMPV and GLMMPV was 0.902 and 1.060 respectively, and the intercept was 2.522 and -1.511 respectively. Furthermore, R^2^ was 0.513 and 0.623 respectively (Fig. 4). These results indicate that GLMMPV was closer to BEPV than PMMPV. Therefore, the GLMM best model may allow more accurate VCM Css-trough predictions.

## Discussion

Figure 4b shows that the simple linear regression slope of BEPV and GLMMPV was closer to 1 than that of BEPV and PMMPV was (Fig. 4a, GLMMPV, 1.060 and PMMPV, 0.902). Additionally, the simple linear regression intercept of BEPV and GLMMPV was closer to 0 than that of the BEPV and PMMPV was (GLMMPV, -1.511 and PMMPV, 2.522). Additionally, because the R^2^ of BEPV and GLMMPV was higher than that of BEPV and PMMPV (GLMMPV, 0.623 and PMMPV, 0.513), we were able to determine that the GLMM best model created in this study predicted the VCM Css-trough with better accuracy than the PMM did for the study subjects. Table 6 shows that the WAIC of the GLMM best model (252.01) was smaller than that of the basic model (258.42, equivalent to the model that predicted Css-trough from the PMM). This indicates that generalization error is decreased in the GLMM best model. Therefore, we believe that the GLMM best model can predict the VCM Css-trough of unknown patients with greater accuracy than the PMM can.

Figure 4 shows that 4.35% (2/46) of patients had PMMPDQ of ≥10 μg/mL, but none had GLMMPDQ of ≥10 μg/mL. Considering the effective blood concentration range of the VCM Css-trough, a difference of ≥ 10 μg/mL in Css-trough predictions would raise concerns that the drug may be less effective and cause adverse effects. However, we believe that the GLMM best model controls large prediction deviations like this.

**Fig. 5:**
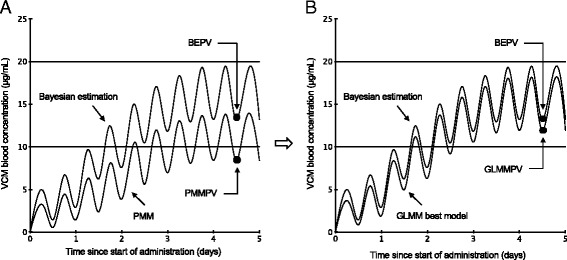
Blood concentration of VCM-time profiles in patient who was benefited more from GLMM best model than from PMM. Patient b in Fig. 4 showed BEPV of 13.20 μg/mL, PMMPV of 8.40 μg/mL, and GLMMPV of 11.93 μg/mL. **a** Patient’s PMMPV showed major differences with the BEPV, putting the level outside the effective blood concentration range. Therefore, PMM led to major disadvantages in prediction. However, the GLMMPV and BEPV in (**b**) were close and effective blood concentration range was reached, indicating the GLMM best model was appropriate for making these predictions

Next, we investigated patients with major improvements in predictive accuracy achieved by changing from the PMM to the GLMM best model in predicting VCM Css-trough. Patient a shown in Fig. 4 had a PMMPDQ and GLMMPDQ of 7.90 and 0.64 μg/mL (the length of the dashed lines in Fig. 4a and b, respectively). Based on this, the change from PMM to the GLMM best model allowed that predictive accuracy was improved 7.26 μg/mL (7.90–0.64 μg/mL). Similarly, patients b and c showed a 3.53 and 3.23 μg/mL (4.80–1.27 and 6.00–2.77 μg/mL) improvement, respectively. The graph of the changes in VCM blood concentration experienced by patient b (Fig. 5a) illustrates that the PMMPV deviated greatly from the BEPV (the absolute PMMPDQ value was large), which caused the blood concentration to fall outside the effective range. However, since the GLMM best model predicted the Css-trough with high accuracy, the GLMMPV was close to the BEPV (the absolute GLMMPDQ value was small) and achieved the effective blood concentration range (Fig. 5b). Similarly, if the Css-trough prediction accuracy can be increased and the achievement of an effective blood concentration range can be accurately predicted when establishing the initial dose plan, then a revised dose plan would be unnecessary. Furthermore, we found that the improvement in the Css-trough prediction accuracy for the patients achieved using the GLMM best model was related to high BUN/adjusted SCr values of these patients (patients a, b, and c: 71.35, 34.52, and 32.17, respectively). It has been reported that when the BUN/SCr is > 20, the estimation of renal function (CLcr) using the CG formula results in overestimations [16]. Therefore, when establishing the initial dose plan using PMM for patients a, b, and c, we assessed the CLcr at a higher than actual level, which led to excessive VCM doses. We speculate that this further caused the deviation between the PMMPV and BEPV. However, when using the GLMM best model we included the BUN/adjusted SCr (fixed effect), which corrected the overestimated renal function in the CG formula and ultimately increased the VCM Css-trough prediction accuracy.

Nevertheless, there were also cases where the GLMM best model created in this study did not improve the predictive accuracy of the Css-trough values. These patients had large deviations (PMMPDQ) between PMMPV and BEPV, and the GLMM best model did not improve the Css-trough prediction accuracy. For example, PMMPDQ and GLMMPDQ of patient d showed large positive deviations (Fig. 4a and b, 11.2 and 9.4 μg/mL, respectively), and those of patient e also showed positive deviations (Fig. 4a and b, 6.9 and 5.5 μg/mL, respectively). PMMPDQ and GLMMPDQ of patient f showed negative deviations (Fig. 4a and b, -7.0 and -8.8 μg/mL, respectively). We believe that these were likely attributable to the effect of changes in SCr after VCM administration commenced. Our results showed that SCr of patient d was 0.60 mg/dL before VCM administration, but rose to 0.85 mg/dL after VCM administration, and SCr of patient e was risen from 1.05 mg/dL to 1.52 mg/dL. We considered that whose renal functions were declined. Our results also showed that SCr of patient f was 1.20 mg/dL before VCM administration, but decreased to 0.82 mg/dL after VCM administration, which we considered that whose renal function was improved. Therefore, since the renal function of these patients changed after VCM administration started (change in CLcr), the Css-trough prediction accuracy worsened, and the absolute PMMPDQ and GLMMPDQ values increased. Furthermore, PMMPDQ and GLMMPDQ of patient g showed large positive deviations (Fig. 4a and b, 8.8 and 6.4 μg/mL, respectively), and those of patient h also showed large positive deviations (Fig. 4a and b, 10.3 and 8.5 μg/mL, respectively). We thought these were due mainly to involvement of hypoalbuminemia. It has been reported that kidney function is overestimated because of proximal tubule secretion of creatinine increases in patients with hypoalbuminemia [17]. The serum albumin levels of patients g and h were 2.4 and 2.0 g/dL, respectively. We considered that overestimation of kidney function in patients g and h led to excessive VCM doses, and rose Css-trough unexpectedly, resulting the absolute PMMPDQ and GLMMPDQ values increased. To solve these problems, new medical data must be extracted and included in the GLMM best model.

## Conclusions

This study demonstrated that the GLMM best model we created for use with the GLMM method in initial VCM dose planning allowed a more accurate Css-trough prediction than PMM did. The GLMM best model increased the rate of achieving the effective VCM blood concentration range. This may lead to reduce the revised dose planning requirement and increase the therapeutic effect of VCM safely.

## References

[1] Sakoulas G, Gold HS, Cohen RA, Venkataraman L, Moellering RC, Eliopoulos GM (2006). Effects of prolonged vancomycin administration on methicillin-resistant Staphylococcus aureus (MRSA) in a patient with recurrent bacteraemia. J Antimicrob Chemother.

[2] Mohr JF, Murray BE (2007). Point: vancomycin is not obsolete for the treatment of infection caused by methicillin-resistant Staphylococcus aureus. Clin Infect Dis.

[3] Jeffres MN, Isakow W, Doherty JS, Micek ST (2007). A retrospective analysis of possible renal toxicity associated with vancomycin in patients with health care-associated methicillin-resistant Staphylococcus aureus pneumonia. Clin Ther.

[4] Lodise TP, Patel N, Lomaestro BM, Rodvold KA, Drusano GL (2009). Relationship between initial vancomycin concentration-time profile and nephrotoxicity among hospitalized patients. Clin Infect Dis.

[5] Ye ZK, Tang H-L, Zhai S-D (2013). Benefits of therapeutic drug monitoring of vancomycin: a systematic review and meta-analysis. PLoS One.

[6] Tsuji Y, Hiraki Y, Mizoguchi A, Sadoh S, Sonemoto E, Kamimura H, Karube Y (2009). Effect of various estimates of renal function on prediction of vancomycin concentration by the population mean and Bayesian methods. J Clin Pharm Ther.

[7] Bakkestuen V, Halvorsen R, Heegaard E (2009). Disentangling complex fine‐scale ecological patterns by path modelling using GLMM and GIS. J Veg Sci.

[8] Jewkes RK, Levin JB, Penn-Kekana LA (2003). Gender inequalities, intimate partner violence and HIV preventive practices: findings of a South African cross-sectional study. Soc Sci Med..

[9] Cockcroft DW, Gault MH (1976). Prediction of creatinine clearance from serum creatinine. Nephron.

[10] Smythe M, Hoffman J, Kizy K, Dmuchowski C (1994). Estimating creatinine clearance in elderly patients with low serum creatinine concentrations. Am J Hosp Pharm.

[11] Yamamoto M, Kuzuya T, Baba H, Yamada K, Nabeshima T (2009). Population pharmacokinetic analysis of vancomycin in patients with gram-positive infections and the influence of infectious disease type. J Clin Pharm Ther.

[12] Guilford JP, Fruchter B (1956). Fundamental statistics in psychology and education.

[13] Watanabe S (2010). Asymptotic equivalence of Bayes cross validation and widely applicable information criterion in singular learning theory. J Mach Learn Res.

[14] Woltman H, Feldstain A, MacKay JC (2012). An introduction to hierarchical linear modeling. Tutor Quant Methods Psychol.

[15] Gelman A, Carlin JB, Stern HS, Dunson DB, Vehtari A, Rubin DB (2013). Bayesian data analysis.

[16] Poggio ED, Nef PC, Wang X, Greene T, Van Lente F, Dennis VW, Hall PM (2005). Performance of the cockcroft-gault and modification of diet in renal disease equations in estimating GFR in Ill hospitalized patients. Am J of Kidney Dis.

[17] Branten AJ, Vervoort G, Wetzels JF (2005). Serum creatinine is a poor marker of GFR in nephrotic syndrome. Nephrol Dial Transplant.

